# Diagnostic Value and Surgical Implications of the 3D DW-SSFP MRI On the Management of Patients with Brachial Plexus Injuries

**DOI:** 10.1038/srep35999

**Published:** 2016-10-26

**Authors:** Ben-Gang Qin, Jian-Tao Yang, Yi Yang, Hong-Gang Wang, Guo Fu, Li-Qiang Gu, Ping Li, Qing-Tang Zhu, Xiao-Lin Liu, Jia-Kai Zhu

**Affiliations:** 1 Department of Microsurgery, Orthopaedic Trauma & Hand Surgery, The First Affiliated Hospital, Sun Yat-sen University, Guangzhou guangdong, 510080, P.R. China

## Abstract

Three-dimensional diffusion-weighted steady-state free precession (3D DW-SSFP) of high-resolution magnetic resonance has emerged as a promising method to visualize the peripheral nerves. In this study, the application value of 3D DW-SSFP brachial plexus imaging in the diagnosis of brachial plexus injury (BPI) was investigated. 33 patients with BPI were prospectively examined using 3D DW-SSFP MR neurography (MRN) of brachial plexus. Results of 3D DW-SSFP MRN were compared with intraoperative findings and measurements of electromyogram (EMG) or somatosensory evoked potentials (SEP) for each injured nerve root. 3D DW-SSFP MRN of brachial plexus has enabled good visualization of the small components of the brachial plexus. The postganglionic section of the brachial plexus was clearly visible in 26 patients, while the preganglionic section of the brachial plexus was clearly visible in 22 patients. Pseudomeningoceles were commonly observed in 23 patients. Others finding of MRN of brachial plexus included spinal cord offset (in 16 patients) and spinal cord deformation (in 6 patients). As for the 3D DW-SSFP MRN diagnosis of preganglionic BPI, the sensitivity, the specificity and the accuracy were respectively 96.8%, 90.29%, and 94.18%. 3D DW-SSFP MRN of brachial plexus improve visualization of brachial plexus and benefit to determine the extent of injury.

Brachial plexus injury (BPI) is the most severe nerve injury of the upper extremities and is often associated with trauma such as, motorcycle accidents^1^. The factors on the restricted recovery of upper limb function, especially those of the hand, after BPI are complicated. Recovery is mainly determined by the interval between injury and treatment times^2^, which in turn depends on early diagnosis. Early reconstructive surgery of brachial plexus injuries encourages rapid regeneration and repair^3^,^4^. However, the early determination of the degree of damage, the time of surgery, and the selection of surgical methods in BPI patients still pose problems. Patients with brachial plexus injuries are typically observed for three to six months to confirm that there is no recovery of function before surgical treatment^5^. However, brachial plexus root avulsions usually require surgical intervention as soon as possible^6^,^7^. A deferred diagnosis misses the best timing for early intervention. Traditionally, brachial plexus injury has been evaluated using clinical history, physical examination, and neurophysiological testing^8^. Neurophysiological tests and physical examination may not reveal the extent of the injury at the early stage and therefore may not supply the guideline of surgery time.

Imaging of the brachial plexus has progressed from plain radiography to CT, and then to CT myelography. Now, CT myelography has gradually been replaced by MR neurography (MRN), which is a non-invasive technique that allows assessment of both the proximal and distal components of the brachial plexus^9^. Since MRN was introduced in 1992^10^, it has gained acceptance as an important diagnostic adjunct to the clinical evaluation of BPI^11^,^12^. The major disadvantage of conventional MRI sequences used to evaluate the brachial plexus was that maximum intensity projection (MIP) can be done but with less image quality^13^. With recent improvements in MRI, especially 3.0-T MRI, images now show 3D high-resolution views of fine structures which plays a vital role in successful preoperative diagnosis of BPI. This in turn is imperative for proper decisions to be made by surgeons^14^. Current state-of-the-art conventional Magnetic Resonance Nerve imaging, involving 2D or 3D high-resolution T1-weighted, (fat-suppressed) T2-weighted, or short TI inversion recovery (STIR) imaging, provides detailed anatomic nerve depiction^15^,^16^. Clinical application of three-dimensional reversed fast imaging by steady-state precession (3D-PSIF) with diffusion-weighted MR sequence showed the successful use in cranial nerve reconstruction in 2008^17^, while a review of the current imaging literature showed rare correlation between MRN results with per-operative findings in BPI. The purpose of this study is to investigate the usefulness of three-dimensional diffusion-weighted steady-state free precession (3D DW SSFP) for BPI diagnosis.

## Materials and Methods

### Patients

Between January 2007 and December 2012, 33 patients with brachial plexus injury (BPI) underwent pre-operative electromyography (EMG) and magnetic resonance imaging (MRI) of the brachial plexus. The actual conditions of the injury were confirmed intra-operatively. The patients included 30 males and 3 females; 21 cases with BPI on the left and 12 on the right; ages between 16 and 52 years old, given a mean age of 33.47 ± 7.41 years old; mean interval between injury and MRI examination of 3.67 ± 1.42 months; reasons for injury: 19 cases of motorcycle accidents, 10 cases of car accidents, 2 cases of falling and 2 cases of mechanical traction injuries. The ethics committee of the First Affiliated Hospital of Sun Yat-sen University approved the study. All methods were carried out in “accordance” with the approved guidelines. All patients gave written informed consent.

### MRI Examination

MRI was performed using a clinical 3.0T MR imager (Magnetom Trio with TIM system, Siemens Medical Systems, Erlangen, Germany) with a standard neck and CP spine array coil. After conventional T1- and T2-weighted spin-echo (SE) imaging was completed, the 3D-PSIF with diffusion-weighted sequence was performed. The patient was supine and the cervical spine was in the conventional position. The magnetic field was centered on the C_6_ vertebra and conventional neck MRI scans were performed. Patients were advised to avoid swallowing as much as possible during the scan and to breathe mainly from the abdomen. If patients had upper limb spasms due to causalgia, appropriate analgesics and sedatives were administered before the examination in order to prevent image interference caused by involuntary limb movements.

The basic parameters of the 3D DW SSFP sequence were as follows: TR 9.26 ms, TE 4.91 ms, matrix: 448 × 448 mm, flip angle, 180°, FOV, 280 × 280 mm; bandwidth, 686 Hz/px; slice thickness, 2 mm, in-plane spatial resolution, 1.1 × 1.5 mm; number of acquisitions, 2; and the acquisition time was 5 minutes 42 seconds.

The 3D DW SSFP sequence was assessed by two experienced radiologists at the same time. MR images were reviewed in a random fashion and all imaging parameters were unknown to the observers.

### Observation and Evaluation

MRI observation and evaluation of normal brachial plexus: the contralateral side was used as a normal reference. The imaging features of the contralateral brachial plexus were observed in all patients with the assistance of a qualified radiologist. All clearly visible intraspinal brachial plexus roots, ganglia, and extraspinal brachial plexus roots, trunks, branches and cords were counted. MRI observation and evaluation of BPI: The MRI features of patient’s BPI were observed and analyzed, followed by comparisons with intraoperative findings and EMG or SEP.

## Results

### Results of MRI observation and evaluation of normal brachial plexus

#### MRI observations of normal brachial plexus

The brachial plexus exhibited high signal intensity in the raw MRI images while the ganglions appeared as hyperintensities. Of the 33 patients in this study, the postganglionic section of the brachial plexus was clearly visible in 26 patients, while the preganglionic section of the brachial plexus was clearly visible in 22 patients. The imaging features were as follows:

#### MRI features of preganglionic brachial plexus

**Figure 1:**
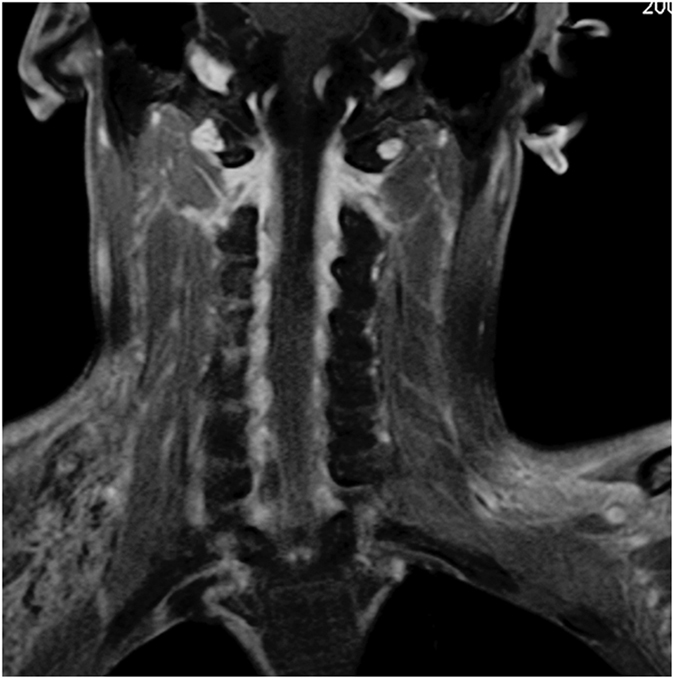
Coronal view of MRI visualizing the ventral rootlets. The number and size of rootlets and the connection with the cord are well visualized.

**Figure 2:**
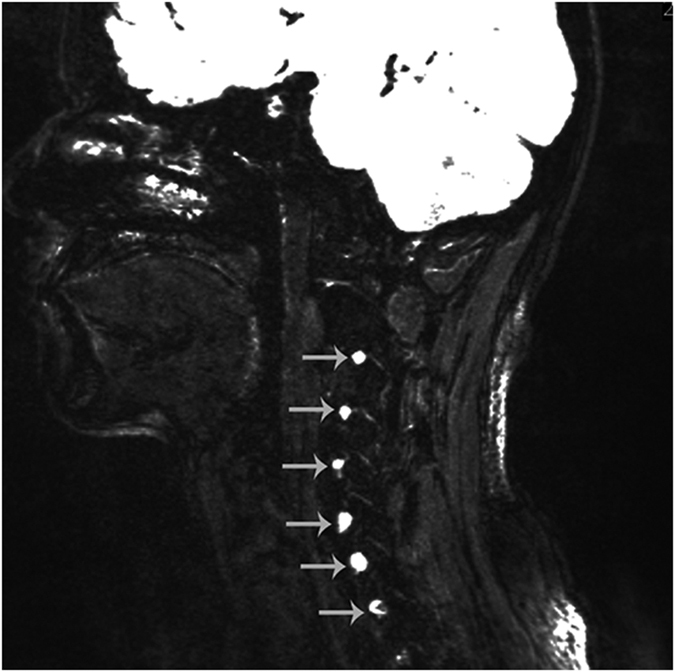
Sagittal view of the MRI, Ganglia C3 through C5 (white arrow) on the right side of the body can be visualized.

The ventral rootlets appeared as low-signal narrow, striped shadows, which swelled to form ganglia near the intervertebral foramen, appearing as hyperintensities. The ventral rootlets could be observed clearly in the coronal view of MRI (Fig. 1), while the ganglia could be observed clearly in sagittal view (Fig. 2).

#### MRI features of postganglionic brachial plexus

Postganglionic brachial plexus appeared as a cord-like hyperintensities structure starting next to the intervertebral foramen between C_5–8_ and T_1_, merging under the clavicle and the axilla. The C_5–8_ and T_1_ nerve roots as well as the superior, middle and inferior trunks formed by their merging were clearly visible. The nerve branches and cords could also be seen, however, due to the higher concentration of anatomical structures, single branches or single cords could not be differentiated.

#### MRI count results of normal brachial plexus

The MRI count results of the 33 patients are shown in Table 1. Visibility of the brachial plexus roots and trunks were the highest, followed by that of ganglia, branches and cords, while that of the intraspinal nerve filaments were the lowest.

### Results of MRI observation and evaluation of BPI

#### MRI Manifestation of Preganglionic BPI

Of the 33 patients, 23 had preganglionic injuries and 10 had postganglionic injuries. The results are as follows.

1. Pseudomeningocele: 51 cysts in 23 patients, the most common feature observed, which appeared as high-density cystic shadows inside and outside intervertebral foramen; 31 left-sided and 20 right-sided. Varied morphology, with the basic shape being triangular.
2. Spinal cord offset: 16 cases; 11 patients shifted toward the injured side and 5 patients shifted toward the contralateral side.
3. Spinal cord deformation: 6 patients. Compression from Pseudomeningocele or traction from avulsed nerve roots led to the loss of a normal oval shape of the spinal in transverse plain.

#### Comparison between MRI and Findings of intra-operative exploration

Among the 33 patients, 103 nerve roots were identified during surgical exploration. 96 roots were positively identified by MRI examination, of which 93 roots were true positives and 3 were false positives. Seven roots were not identified by MRI, of which 3 were false negatives and 4 were true negatives. As for the MRI diagnosis of preganglionic BPI, the sensitivity was 96.8%, specificity was 90.29% and accuracy was 94.18%. Chi-squared tests were used to compare the difference between MRI and findings of intra-operative exploration, *P* = 0.23 > 0.05, hence the difference was not significant (Table 2).

#### MRI Manifestation of Postganglionic BPI

**Figure 3:**
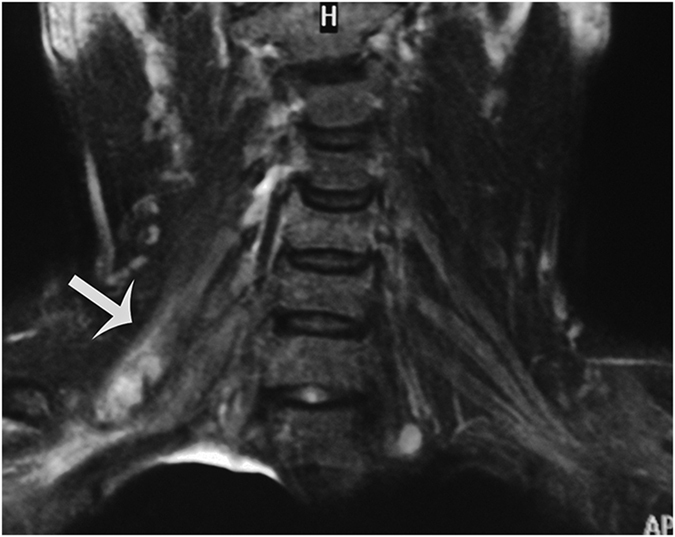
A 27-year-old man with history of fall injury three months back. Coronal image shows discontinuity of C5, retraction of distal nerves after rupture, disappearance of local nerve structure, replacement by scar tissue (white arrow).

**Figure 4:**
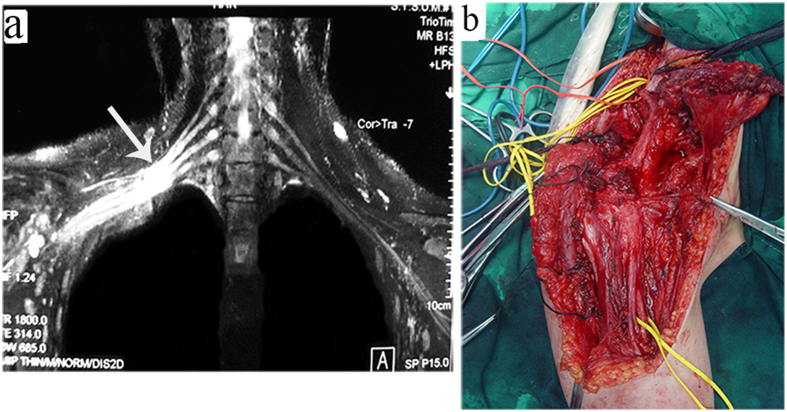
An 18-year-old female with a history of stretch injury by machine. (**a**) Coronal image shows disappearance of local nerve structure, replacement by scar tissue or hematoma, and neural appearance could not be observed on MRI at the level of the trunks and cords. (**b**) Accompanying clinical picture shows as figure a at the same level and surrounding scarring. a long segment of scar tissue surrounding the nerves and nerve defect no more than 20 cm.

**Figure 5:**
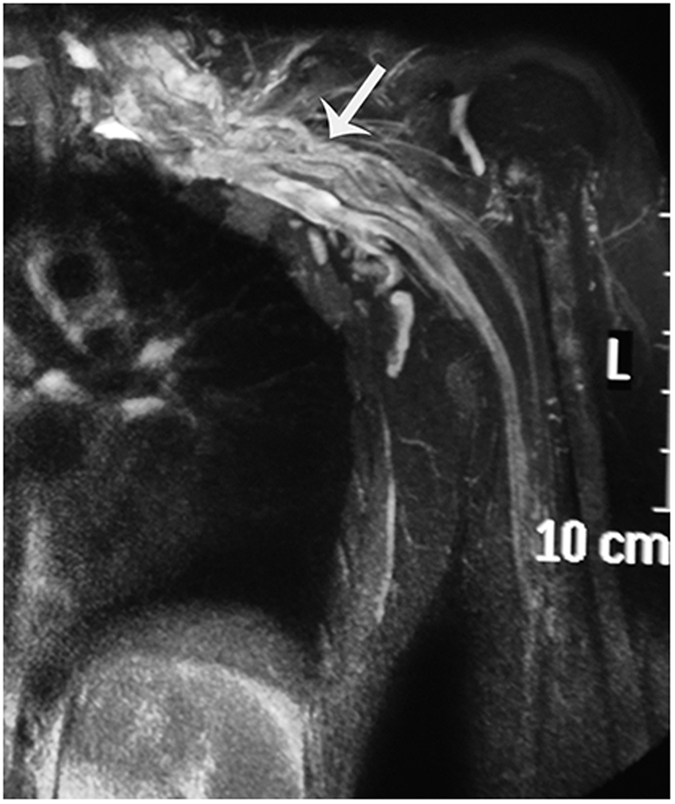
A 27-year-old man with a history of motorcycle accident one month back. Coronal image shows loss of normal smooth neural pathways, meandering or even curling pathways from root to cord.

Of the 33 patients, postganglionic brachial plexus was clearly visible in 26. The results of observation are as follows:

1. Nerve discontinuity, retraction of distal nerves after rupture, disappearance of local nerve structure, replacement by scar tissue or hematoma, and neural appearance could not be observed on MRI (Fig. 3).
2. Abnormal neural signals often manifested as hyperintensities T_2_-weighted images, significant neural thickening, and unclear boundaries between thickened nerves (Fig. 4a).
3. Abnormal shapes in neural pathways, loss of normal smooth neural pathways, meandering or even curling pathways (Fig. 5).
4. Abnormal signals from soft tissues surrounding the damaged nerve, including (1) scarring of the anterior scalene muscle, (2) local scarring around damage nerves, appeared as hyperintensities in T_2_ images.

## Discussion

### Anatomy of the brachial plexus

The brachial plexus innervates the muscles, joints and skin of the shoulder and upper limb. It is formed by the ventral rami of C5–C8 and T1 nerves. There is irregular contribution from the C4 and T2 rami in a pre or post fixed plexus, respectively. The C5 and C6 roots join to form the superior trunk, the C7 root forms the middle trunk and C8 and T1 roots join to form the inferior trunk. Each trunk gives anterior and posterior divisions. The posterior divisions of all the three trunks join to form the posterior cord. The anterior divisions of superior and middle trunks form the lateral cord. The anterior division of the inferior trunk forms the medial cord. The axillary and radial nerves are the terminal branches of the posterior cord. The musculocutaneous nerve and lateral root of the median nerve arise from the lateral cord. The medial root of median nerve, ulnar nerve and medial cutaneous nerves in the arm and forearm arise from the medial cord^18^.

### 3D-imaging technology of MRN

The MRN protocol includes a combination of T1W, T2W, STIR, 3D STIR SPACE and 3D T2 SPACE sequences. Recently, the integration of peripheral nerve imaging sequences and post-processing 3D-reconstruction technology has become the focus of research. In 2007, Wolfgang *et al*.^19^ reported the successful reconstruction of healthy human sciatic nerve using multi-planar 3D reconstruction. Viallon *et al*.^20^ used an isotropic 3D T2 Short Term Inversion Recovery (STIR) sequence to image normal brachial plexus and brachial plexus tumors and visualize the relationship between the brachial plexus tumors and nerves. P. Mürtza^21^ reported that Diffusion-weighted MRN is superior for lumbosacral plexus, but not for brachial plexus. Zhang *et al*.^22^ designed a sequence based on 3D DW-SSFP which was able to visualize the main trunk and branches of the cranial nerve. Compared to other gradient echo sequences, the echo signals of 3D DW-SSFP are produced by radio frequency (RF) excitation, which has obvious T2 contrast characteristics. As the echo signals are produced by RF excitation, the spin relaxation is less affected by inhomogeneity in the magnetic field. Thus, the advantages of 3D DW-SSFP in neuroimaging are as follows: firstly, the echo signals obtained by the gradient echo using this sequence has T2 contrast characteristics which can achieve sufficiently high resolution within a reasonable scan time while relatively less affected by inhomogeneity within the magnetic field. Furthermore, by applying water excitation for fat suppression, it is less affected by chemical shift, while the application of the diffusion gradient method allows for effective fluid suppression^23^. Secondly, the sequence involves 3D Fourier imaging, which allows us to obtain any level of image after various post-processing of the raw image. In addition, as the SSFP signal requires certain steady-state conditions, the proton spin of motion, such as that of blood flow, will be manifested as signal loss^24^, which is extremely important in the imaging of small nerves, especially when they are located among larger surrounding blood vessels.

We attempted to use this sequence in the imaging of the brachial plexus and obtained clear images showing the various sections of the brachial plexus with stable imaging in this study.

### MRI features of BPI and their clinical significance

#### Pre-ganglionic injuries

In pre-ganglionic injuries, approximately 20% of the patients show altered signal intensity in the spinal cord on the affected side due to edema, hemorrhage or myelomalacia^25^. In this study pseudomeningocele is the most common finding in MRI. It is an indirect sign of preganglionic injury, well appreciated on T2 WI. The formation mechanism is the outflow of cerebrospinal fluid due to avulsion of the nerve sheath tumor, indicating the presence of nerve root avulsion^26^. Pseudomeningoceles may occur alone without root avulsion in 15% of the cases, and conversely, 20% of nerve root avulsions will not have a pseudomeningocele^27^. In this study, 5 cases with nerve root avulsions did not have a pseudomeningocele and 8 cases without pseudomeningocele proved to be root avulsion by intra-operative neurophysiological testing. Other signs of root avulsion in this study include enhancement of intradural nerve roots or root stumps. This feature was not only found on plane of the nerve root injury, it was also found in adjacent uninjured planes, which might have been due to the outward traction of the brachial plexus during injury.

**Figure 6:**
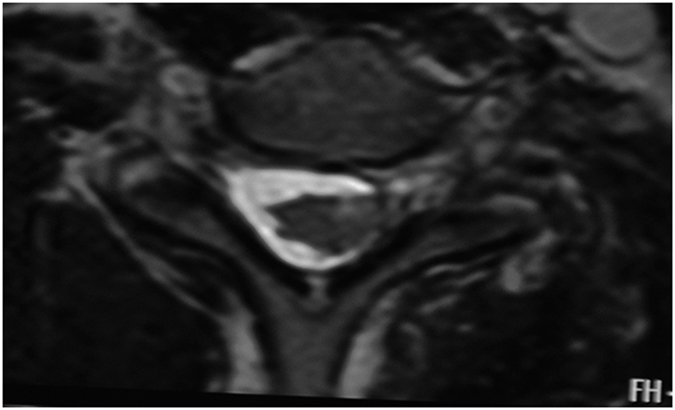
A 25-year-old man with a history of road traffic accident three months back. Axial T2-Wimage showing spinal cord displaced injured side at C6 level; suggesting partial rupture of spinal cord ligament.

Direct visualization of the intradural nerve roots is not always adequate, with reported accurate diagnosis in 52% of cases^28^. Obvious loss of ganglia compared to the contralateral side is mostly observed on the coronal view. Normal ganglia are manifested as round areas with high signal intensities in MRI. Their diameters are slightly larger than those of nerve roots and multiple ganglia are arranged longitudinally. After BPI the features disappeared on the affected side, while there was hyperintensities over a larger area at the ganglia compared to that on the unaffected side. Another indirect sign of preganglionic injury was spinal cord signal intensity changes^29^ which is observed in approximately 20% of patients with preganglionic injuries. We found spinal cord offset and deformation in 8 cases. The spinal cord could be toward the injured or contralateral side. The mechanism is due to the rupture of nerve roots or cord ligament after violent avulsion of the spine. Complete rupture will cause a shift towards the contralateral side, while a partial rupture will cause a shift to the injured side (Fig. 6). The mechanism of spinal cord deformation might be due to the compression of local meningeal cysts.

#### Post-ganglionic injuries

**Figure 7:**
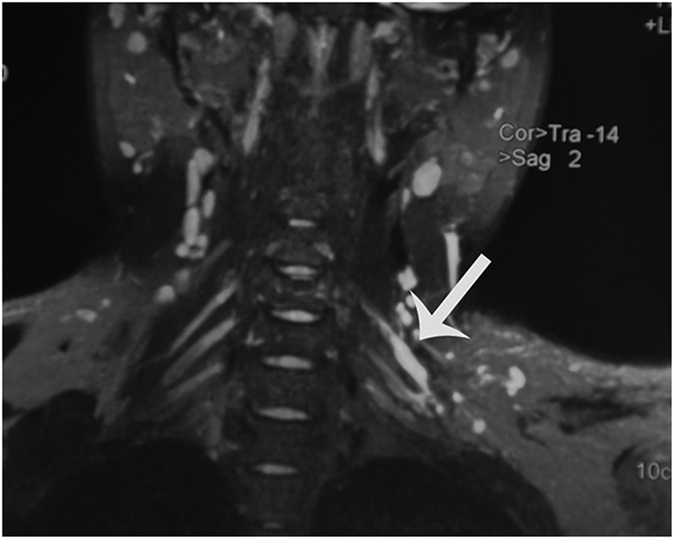
A 25-year-old man with a history of motor cycle accident nine months back. Coronal image shows C5 root is continuous but thickening in T2-weighted images.

The post-ganglionic plexus can be evaluated by MRI with findings suggesting neuropraxia, axonotmesis or neurotmesis^30^. Nerve discontinuity on MRI implied retraction of distal nerves after rupture, disappearance of local nerve structure, and replacement by scar tissue or hematoma. If these features are discovered at the early stage, immediate surgical examination will be needed and the majority of injury nerves can be directly sutured. However, if discovered slightly later, there will be heavy local scarring that is difficult to remove surgically. The distance of nerve retraction is also longer and difficult to be sutured directly, therefore it should be repaired by nerve transfer. Neural thickening and nerve continuity was still present but often manifested as hyperintensities in T2-weighted images (Fig. 7). Nerves on the injured side were obviously thicker than those on the contralateral side, while the relationship between thickened nerves was unclear. Whether surgical intervention or more conservative therapies is appropriate depended on EMG or SEP. If the EMG and SEP are positive, neurolysis would be performed, otherwise the nerve transfer would be the method used. Of the 33 patients in this study, 6 cases exhibited such manifestations. During surgical examination it was found that the nerves had slightly harder texture and electrical stimulation of the lesion caused muscle contraction, therefore only neurolysis was performed. High hyperintense signal changes of flat and long segments in T2W images implied that the differentiation between nerves and surrounding soft tissues were unclear. Such manifestations indicated more severe conditions than root avulsions and treatment was more difficult. Intraoperative examination revealed a long segment of scar tissue surrounding the nerves where the nerve defect was no more than 20 cm, and then left rare reconstructive options, such as contralateral C7 (CC7) and vascularized ulnar nerve transfer (Fig. 4b).

### Effects of brachial plexus MRI on surgical selection in BPI

If MRI examinations are carried out at the early stages of BPI, which can elucidate the status of nerve damage, surgical interventions can be done at the early stage. Not only can brachial plexus MRI be used to identify preganglionic and postganglionic damage, it can also be used to localize postganglionic damage, thus providing a reference for surgical incisions. Therefore, it is imperative that patients with suspected BPI should first undergo MRI examination as soon as possible. Patients who had BPI clinical manifestation with MRI showing the continuous nerve survival and without abnormal signals of the brachial plexus when compared to the unaffected side can receive conservative treatment for three months.

Patients with the following conditions should immediately receive surgery: 1. Medical history of penetration wound with clinical manifestation of BPI, and nerve discontinuity revealed in MRI; 2. MRI reveals widespread abnormal brachial plexus signals, with unclear boundaries between nerves and surrounding soft tissues; 3. MRI indicates brachial plexus root avulsion.

## Limitations

There are certain limitations to the study. One of the limitations is the small sample size. Second, it takes 30 minutes to 40 minutes to complete the examination which might increase the chance of motion artifact, especially for patients with claustrophobia. Third, division and cord segments of the brachial plexus gather into a bundle therefore, it is difficult to distinguish each nerve. Forth, due to the factors such as the cerebrospinal fluid and breathing, there is a certain rate of false positives (13%). Finally, cost might be a major problem limiting the common use of brachial plexus MRI examination.

## Conclusion

Three-dimensional diffusion-weighted steady-state free precession (3D DW-SSFP MRN) improve visualization of brachial plexus from root to branch and help to determine the level of the injury and character of injury. 3D DW-SSFP MRN was demonstrated to be useful in preoperative diagnoses of the type, presurgical planning and timing of surgery for brachial plexus injuries.

## Additional Information

**How to cite this article**: Qin, B.-G. *et al*. Diagnostic Value and Surgical Implications of the 3D DW-SSFP MRI On the Management of Patients with Brachial Plexus Injuries. *Sci. Rep.* **6**, 35999; doi: 10.1038/srep35999 (2016).

**Publisher’s note:** Springer Nature remains neutral with regard to jurisdictional claims in published maps and institutional affiliations.

## Figures and Tables

**Table 1:** Count results of normal brachial plexus.

Brachial plexus position	Count results
Intraspinal nerve filament	21/33(63.64%)
Ganglion	28/33(84.85%)
Brachial plexus root	30/33(90.91%)
Brachial plexus trunk	30/33(90.91%)
Brachial plexus branch and cord	26/33(78.79%)

**Table 2:** Comparison of MRI and Findings of intra-operative exploration, p = 0.23 > 0.05.

		Results of MRI Examination		Total
		+	−	
Findings of intra-operative exploration	+	93	3	96
	−	3	4	7
Total		96	7	103

Data were presented as Chi-squared tests.
